# Large cell neuroendocrine carcinoma presenting with neck swelling in the submandibular gland: a case report

**DOI:** 10.1186/1752-1947-7-81

**Published:** 2013-03-19

**Authors:** Hideto Kawaratani, Tatsuhiro Tsujimoto, Masaaki Yoshikawa, Fumi Kawanami, Yusaku Shirai, Hitoshi Yoshiji, Kousaku Morita, Hiroshi Fukui

**Affiliations:** 1 Third Department of Internal Medicine, Nara Medical University, 840 Shijo-cho, Kashihara, Nara 634-8522, Japan; 2 Department of Diagnostic Pathology, Nara Medical University, 840 Shijo-cho, Kashihara, Nara 634-8522, Japan

**Keywords:** Autopsy, Large cell neuroendocrine carcinoma, Salivary gland, Submandibular gland

## Abstract

**Introduction:**

Large cell neuroendocrine carcinoma in the salivary glands is rare. We report a second case of large cell neuroendocrine carcinoma of the submandibular gland diagnosed at autopsy, and a review of the literature.

**Case presentation:**

A 68-year-old Japanese man was referred to our hospital for thorough investigation of swelling on the right side of his neck. Fine-needle aspiration cytology of the cervical mass suggested poorly differentiated metastatic carcinoma. The primary tumor could not be detected by several examinations. One month after admission, he died of cancer. An autopsy was performed, and it revealed a tumor of the right submandibular gland. The histopathological diagnosis was large cell neuroendocrine carcinoma of the submandibular gland.

**Conclusion:**

To the best of our knowledge, only eight cases of large cell neuroendocrine carcinoma in the salivary glands, including our case, have been reported. This report indicates total biopsy and immunohistochemistry are necessary for diagnosing large cell neuroendocrine carcinoma properly.

## Introduction

Large cell neuroendocrine carcinoma is defined as a variant form of large cell carcinoma, and is classified in the wide spectrum of primary neuroendocrine tumors together with small cell carcinoma (SCC) and atypical carcinoid tumor. Travis *et al*. proposed a distinctive clinicopathological entity of pulmonary neuroendocrine tumors in 1991 [1]. Large cell neuroendocrine carcinoma has poor prognosis like SCC. Although the lung is the most common location of this tumor, large cell neuroendocrine carcinomas have been found in other organs, including the thymus [2], stomach [3], gall bladder [4], uterine cervix [5], urinary bladder, and so on. In the salivary glands, neuroendocrine carcinoma is sometimes seen [6], but large cell neuroendocrine carcinoma is extremely rare, and only seven cases have been reported [7-12]. Their biological behaviors are still unknown. Recently, we have encountered large cell neuroendocrine carcinoma of the submandibular gland that was diagnosed at autopsy, but was difficult to distinguish from metastatic tumor. Here we describe this rare case and present a review of the literature.

## Case presentation

**Figure 1:**
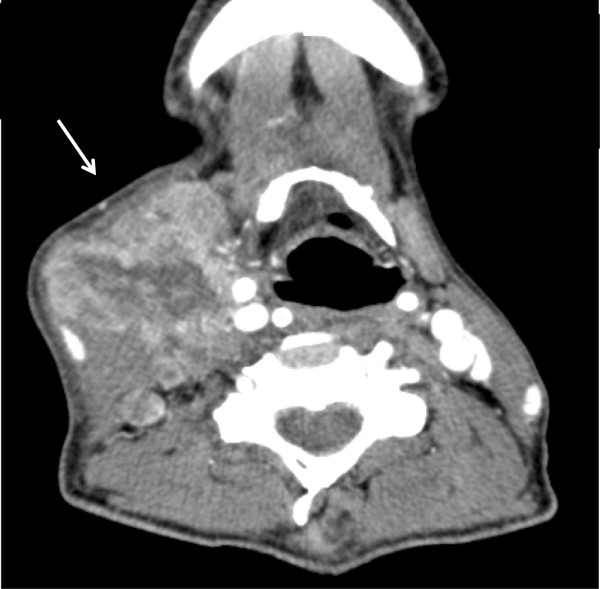
Computed tomography showed enlargement of lymph nodes on the right side of the neck (arrow).

A 68-year-old Japanese man was referred to our hospital for thorough investigation of right hypochondriac pain and painless swelling on the right side of his neck. He had a past history of a transverse colon cancer operation about 18 years earlier and underwent distal gastrectomy and cholecystectomy due to duodenal ulcer 30 years earlier. He had gone to another hospital for diabetes mellitus follow up. One month earlier, he was referred to the same hospital because of right hypochondriac pain and anorexia. Plain abdominal computed tomography (CT) scanning revealed a low density area in his liver segment 8. At first, because of his cervical swelling, hepatic carcinoma with neck metastasis was suspected. He was admitted into an affiliated hospital for further examinations. Esophagogastroduodenoscopy and colonoscopy were performed, but both revealed no malignancy. Enhanced abdominal CT scanning showed no tumor in his liver and other abdominal organs. Enhanced thoracic CT scanning showed enlargement of right cervical lymph nodes (Figure 1), but no primary tumors could be detected. ^18^F-fluorodeoxyglucose (FDG) positron emission tomography (PET) could not be taken because of the patient’s poor systemic condition and there was no instrument in our hospital. Subsequently, fine-needle aspiration cytology (FNAC) of the right cervical mass was performed, and it suggested poorly differentiated carcinoma. Metastatic carcinoma was suspected, but primary organs could not be detected. For further examinations, he was transferred to our hospital after 3 weeks. He had a poor systemic condition and showed multiple metastases of the spine. Then, disseminated intravascular coagulation gradually developed. He died of cancer 5 days after his transfer to our hospital.

**Figure 2:**
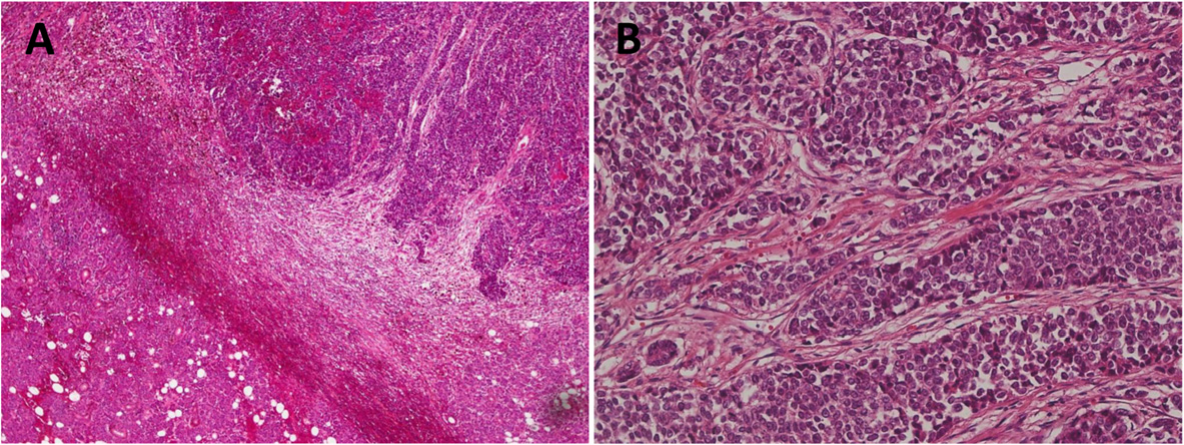
**Hematoxylin and eosin staining showed solid growth of large polygonal atypical cells, organoid structure, palisading, and rosette growth. A**: 40-fold, **B**: 200-fold.

**Figure 3:**
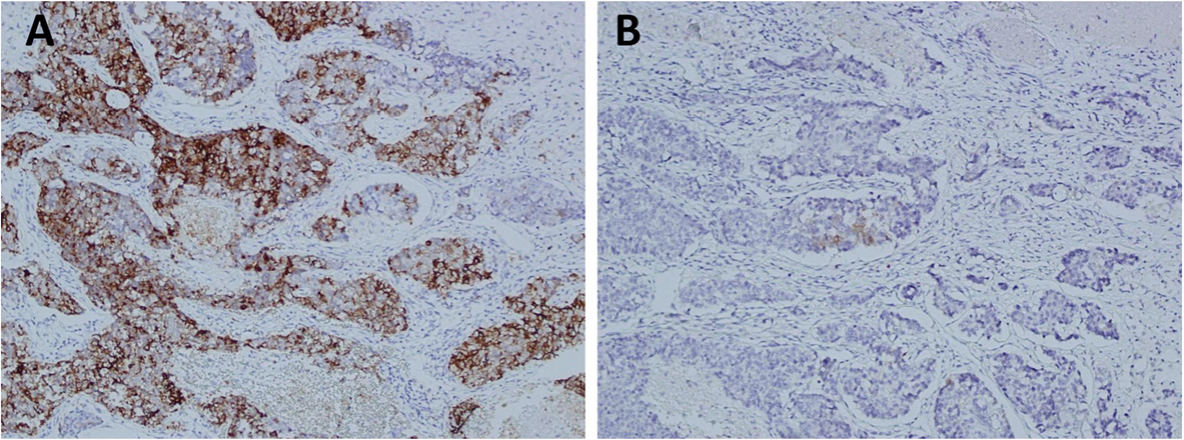
**A: On immunohistochemistry, the tumor cells show positive for CD56. B**: The tumor cells show positive for synaptophysin.

Because the primary carcinoma was unknown, autopsy was performed after his death. At autopsy, a tumor of about 5cm was found in the right submandibular gland. No other primary malignant tumor was detected, except metastasis to the bone marrow and spine. Histological examination of the submandibular tumor revealed a solid growth formed of large polygonal atypical cells. An organoid structure, and palisading, rosette growth were seen, and the tumor had focal squamous differentiation (Figure 2). The tumor showed diffuse necrosis and many mitoses (about 40 cells/10 high-power field). It was difficult to distinguish poorly differentiated SCC, basal cell adenocarcinoma, mixed SCC, and basaloid SCC. Immunohistochemically, CD56 and synaptophysin were positive (Figure 3), whereas chromogranin A, p63, alpha smooth muscle actin, and thyroid transcription factor-1 were negative. There is no report of salivary basaloid SCC. And basal cell adenocarcinoma or mixed SCC were ruled out because of positive synaptophysin which is expressed on neuroendocrine tumors. Based on these findings, the tumor was finally diagnosed as large cell neuroendocrine carcinoma of the submandibular gland.

## Discussion

Salivary gland carcinoma is a rare neoplasm accounting for 0.3% of all human cancers. The treatment for salivary gland carcinoma is mainly surgery, including primary tumor resection with or without neck lymph node dissection and adjuvant therapy. Salivary gland carcinoma is mainly diagnosed by CT or MRI and echo-guided FNAC. Recently, FDG-PET has become a useful modality for detecting salivary gland carcinoma. And FDG-PET has been reported to be superior to CT and/or MRI in detecting neck cancers [13].

Large cell neuroendocrine carcinoma is a poorly differentiated, high-grade neuroendocrine neoplasm that has several morphological and biological features between SCC and atypical carcinoid [1]. The lung is the most common location of large cell neuroendocrine carcinoma. The main features of large cell neuroendocrine carcinoma are those of neuroendocrine cancer, such as organoid nesting, palisading, rosette trabeculae, large cells with a polygonal shape, a relatively low nuclear to cytoplasmic ratio, frequent necrosis mimicking non-SCC, and positive neuroendocrine immunohistochemical markers. To confirm the neuroendocrine features, the tumor cells were stained with antibodies to CD56, synaptophysin, and chromogranin A [14]. Recently, various immunohistochemical techniques have been performed to diagnose neuroendocrine tumors, like neural cell adhesion molecule [15].

This tumor is very rare, and hence there is no consensus on management guidelines. As large cell neuroendocrine carcinoma is sometimes mistaken for a poorly differentiated carcinoma or SCC, immunohistochemistry is important in making the diagnosis of large cell neuroendocrine carcinoma. For pulmonary large cell neuroendocrine carcinoma, surgery is the main treatment and can be complemented with postoperative radiotherapy or chemotherapy. The prognosis of pulmonary large cell neuroendocrine carcinoma is poor. The prognosis of salivary gland large cell neuroendocrine carcinoma seems to be poor, too. Early diagnosis and early treatment are desirable for improvement of the outcomes. More studies are needed to better define the therapeutic alternatives and prognostic factors of salivary gland large cell neuroendocrine carcinoma.

Our review of the medical literature in PubMed between 1975 and 2011 revealed seven cases of salivary gland large cell neuroendocrine carcinoma [7-12]. The details of all eight reported cases, including our present case, are shown in Table 1. There were five men and two women, and their mean age was 75 years (ranging from 68 to 88 years). The most commonly presenting symptom was painless swelling of the neck. The location of the tumors was: five in the parotid and two in the submandibular and none in the sublingual gland. Moreover, five were on the right side and two were on the left side. FNAC was performed in five cases. The pathological findings of FNAC were undifferentiated or poorly differentiated carcinoma. The size varied greatly from 2cm to 9cm. Synaptophysin was positive in six cases, and chromogranin A was positive in three cases. Five cases underwent surgery and radiation therapy postoperatively. One case underwent a combination of chemotherapy and radiation therapy. The outcome varied; three cases died after surgery (5 months, 8 months, and 4 years), whereas three cases had a good outcome with improvement of tumor and free from tumor recurrence after treatment (8 months, 27 months, and 3 years). In our case, the patient died of cancer, and the autopsy revealed large cell neuroendocrine carcinoma of the submandibular gland.

**Table 1:** Previously reported cases of salivary gland large cell neuroendocrine carcinoma

**Reference**	**Age**	**Sex**	**Past history**	**Presenting complaints**	**Size of tumor**	**Location**	**FNAC findings**	**Immunohisto-chemical findings**	**Treatment**	**Outcome**
Hui *et al.*[7]	n.r.	n.r.	n.r.	n.r.	n.r.	n.r.	not performed	n.r.	n.r.	n.r.
Larsson and Donner [8]	88	F	n.r.	mobile mass of neck	2cm	right parotid	undifferentiated carcinoma	Chr (−), Syn (+)	Op and Rx	3 years disease free
Nagao *et al.*[9]	72	F	n.r.	enlargement of neck	7cm	right parotid	large cell undifferentiated carcinoma	Chr (−), Syn (+)	Cx and Op and Rx	died 5 months after operation
Nagao *et al.*[9]*.*	73	M	n.r.	enlargement of neck	3.3cm	right parotid	not performed	Chr (+), Syn (−)	Op and Rx	died 4 years after operation
Casas *et al.*[10]	74	M	n.r.	enlargement of neck	9cm	left parotid	large cell undifferentiated carcinoma	Chr (+), Syn (+)	Op and Rx	8 months disease free
Ueo *et al.*[11]	72	M	gastric cancer	enlargement of neck	4.5cm	right parotid	not performed	Chr (+), Syn (+)	Op and Rx	died 8 months after operation
Sowerby *et al.*[12]	81	M	none	facial paralysis	6cm	left submandibular	atypical lymphoid proliferation	Chr (−), Syn (+)	Cx and Rx	27 months disease free
This report	68	M	DM, colon cancer	abdominal pain enlargement of neck	5cm	right submandibular	poorly differentiated carcinoma	Chr (−), Syn (+)	none	died 1 month after symptom appeared

Chr = chromogranin; Cx = chemotherapy; DM = diabetes mellitus; FNAC = fine-needle aspiration cytology; n.r. = not recorded; Op = operation; Rx = radiation; Syn = synaptophysin.

## Conclusion

In this report we presented a rare case of large cell neuroendocrine carcinoma of the submandibular gland. Diagnosis based on FNAC findings was difficult because of the small samples of tissue. If a patient presents with enlargement of the neck, then total biopsy or operation and immunohistochemistry are needed to diagnose salivary gland tumor. Since salivary gland large cell neuroendocrine carcinoma is rare and has a poor prognosis, similar to the pulmonary large cell neuroendocrine carcinoma, early diagnosis is necessary to improve the prognosis of this disease.

## Consent

Written informed consent was obtained from the patient’s family for publication of this case report and accompanying images. A copy of the written consent is available for review by the Editor-in-Chief of this journal.

## Competing interests

All authors declare that they have no competing interest.

## Authors’ contributions

HK analyzed and interpreted the patient data. MY and FK have been involved in direct patient care at different time points. YS did the literature search. KM performed the pathological examination of the tumor. TT and HY were major contributors in writing the manuscript. HF did the final draft proof reading and approval. All authors read and approved the final manuscript.
